# Effect of butorphanol, midazolam or ketamine on romifidine based sedation in horses during standing cheek tooth removal

**DOI:** 10.1186/s12917-017-1299-6

**Published:** 2017-12-06

**Authors:** Theresa Maria Müller, Klaus Hopster, Astrid Bienert-Zeit, Karl Rohn, Sabine B.R. Kästner

**Affiliations:** 1 Clinic for Horses, University of Veterinary Medicine Hannover, Foundation, Bünteweg 9, 30559 Hannover, Germany; 2 Institute for Biometry, Epidemiology and Information Processing, University of Veterinary Medicine Hannover, Foundation, Bünteweg 9, 30559 Hannover, Germany

**Keywords:** Horse, Romifidine, Sedation, Midazolam, Ketamine, Cheek tooth removal

## Abstract

**Background:**

Standing surgery, especially dental procedures, are commonly performed in horses. This leads to an increasing demand for reliable sedation protocols. Therefore, it was the purpose of this study to investigate the influence of butorphanol, midazolam or ketamine on romifidine based sedation in horses during cheek tooth removal.

**Methods:**

Forty horses presented for tooth extraction were divided in four groups using matched pair randomization. Group R was sedated with romifidine (bolus 0.03 mg/kg, followed by a constant rate infusion (CRI) 0.05 mg/kg/h) and group RB with romifidine (same dose) and butorphanol (0.02 mg/kg; CRI 0.04 mg/kg/h). Group RM received romifidine (same dose) and midazolam (0.02 mg/kg; CRI 0.06 mg/kg/h) whereas group RK was administered romifidine (same dose) and ketamine (0.5 mg/kg; CRI 1.2 mg/kg/h). If sedation was not adequate a top up bolus of romifidine (0.01 mg/kg) was administered. The quality of sedation and the conditions for tooth extraction, the level of ataxia, chewing, head and tongue movement were evaluated by using a scoring system. The investigator was blinded to the applied sedation protocol. Furthermore, serum cortisol concentrations before, during and after the procedure were analyzed to gain more information about the stress level of the horses.

**Results:**

Horses in group RM showed significantly less chewing and tongue activity compared to horses sedated with romifidine alone or with butorphanol additionally, but also significantly higher levels of ataxia. The quality of sedation was significantly better if romifidine was administered in combination with ketamine compared to romifidine alone. Furthermore, horses of group RK needed less additional romifidine boli compared to all other groups. Blood cortisol concentrations during surgery in groups RB and RM remained unchanged. Horses of group R showed higher cortisol concentrations during sedation compared to horses of groups RB and RM.

**Conclusion:**

Romifidine alone at an initial bolus dose of 0.03 mg/kg followed by a constant rate infusion of 0.05 mg/kg/h was insufficient to obtain an adequate level of sedation and led to increased stress levels, whereas the addition of butorphanol inhibited the stress response. The combination of romifidine with either midazolam or ketamine improved sedation quality and surgical conditions.

## Background

An increasing number of surgical procedures are conducted under standing sedation in horses [1]. Chemical restraint is usually obtained with a sedative often in combination with an opioid [2]. Bolus-administration followed by constant rate infusion (CRI) or repeated injections of boli are possible application methods. A CRI has the advantage of achieving a continuous plasma level and obtaining a constant depth of sedation [3], thereby reducing the adverse effects on the cardiovascular system [4].

Alpha-2 agonists are the most frequently utilized sedatives in equine medicine. Within this group romifidine achieves the longest duration of sedation [5]. Although the longer lasting sedative effect seems disadvantageous for an infusion, this compound produces the least ataxia of the currently available alpha-2 agonists [5]. This could be a major advantage for surgical procedures in the standing horse and outweighs the lower controllability of romifidine administered as CRI. The combination of opioids with sedatives induces a deeper and more reliable sedation than either drug alone [2]. The use of opioids alone is limited by the risk of inducing central stimulation and excitement, which might also contribute to “head bobbing” that is sometimes seen in horses after sedative combinations including opioids [6]. The agonistic activity of butorphanol at ĸ-opioid receptors as well as its competitive antagonist and agonist activity at μ- and δ-opioid receptors exerts the analgesic effect [7].

The benzodiazepine midazolam mediates muscle relaxant and anticonvulsant effects by its agonistic action on the inhibitory GABA_A_-receptor in the brainstem, reticular formation and spinal cord [8]. Midazolam produces no sedative effect in adult horses [9]. Advantages of midazolam for dental procedures in horses have been described [10], but higher degrees of ataxia can occur [9, 10].

Ketamine is commonly used for dissociative anaesthesia in horses [11]. Recent studies showed beneficial analgesic potential of low dose ketamine in the standing horse [10, 12] without any sedative effect [13].

Cortisol concentrations can be used to quantify stress levels in humans and animals [14–16]. Physical restraint, manipulations in the mouth and pain represent potential stress-inducing factors for animals [15, 17]. All of these do occur during cheek tooth extractions. Therefore, plasma cortisol concentrations could be used as an indicator of insufficient sedation during surgery.

The aim of this study was to find a romifidine based sedation protocol for dental procedures, which produces reliable sedation allowing tooth extraction and minimizes stress reactions in horses.

## Methods

### Study design

Prospective clinical trial.

### Animals

The study included 40 horses presented for cheek tooth extraction one to 2 days prior to surgery at the Clinic for Horses, University of Veterinary Medicine Hannover, Foundation. Horse owners gave informed consent for their animals’ inclusion in the study and the study was approved by the institutional ethical committee of the University of Veterinary Medicine in Hannover, Germany. All horses were considered systemically healthy based on clinical examination. In the clinic they were fed hay and free access to water was provided.

### Preparation

Three hours prior to sedation a 12-G intravenous catheter (INTRAFLON 2®)^1^ was placed aseptically into the left or right jugular vein and meloxicam 0.6 mg/kg bwt (Metacam® 20 mg/ml)^2^ was administered intravenously (i.v.). Neither food nor water was withheld prior to sedation. Horses were placed in stocks and left unhandled for 10 min for acclimatization. Self-adhesive electrode pads were attached to the thoracic wall of the horses to monitor the heart rate (HR) during the procedure via an electrocardiogram (Televet 100 - Telemetric ECG & Holter).^3^ The respiratory rate (RR) was recorded based on visual observation. The relative head height was determined by measuring the distance from the rostral end of the nostrils to the ground.

### Sedation protocols

Matched pair randomization was used to assign the horses to one of four different sedation protocol groups, thereby assuring proper homogeneity and comparability between groups. For example, if an 18-year-old Icelandic horse was assigned to group R, the next Icelandic horse or horse of the same age group was assigned to one of the three remaining groups. This procedure was continued until all four groups were assigned.

Assigned treatment groups were:

- Group R: Bolus romifidine (Sedivet® 10 mg/ml)^2^ 0.03 mg/kg bwt i.v., followed by CRI romifidine 0.05 mg/kg bwt/h i.v.
- Group RB: Bolus romifidine 0.03 mg/kg bwt and butorphanol (Alvegesic® vet. 10 mg/ml)^4^ 0.02 mg/kg bwt i.v., followed by CRI romifidine 0.05 mg/kg bwt/h and butorphanol 0.04 mg/kg bwt/h i.v.
- Group RM: Bolus romifidine 0.03 mg/kg bwt i.v. and midazolam (Midazolam B. Braun 5 mg/ml)^5^ 0.02 mg/kg bwt i.v., followed by CRI romifidine 0.05 mg/kg bwt/h and midazolam 0.06 mg/kg bwt/h i.v.
- Group RK: Bolus romifidine 0.03 mg/kg bwt and ketamine (Narketan® 100 mg/ml)^6^ 0.5 mg/kg bwt i.v., followed by CRI romifidine 0.05 mg/kg bwt/h and ketamine 1.2 mg/kg bwt/h i.v.

Drug dosages were based on a previous study from Hopster et al. [10]. The initial drug bolus was diluted in physiologic saline to a volume of 100 ml and administered over 10 min. The CRI medication was mixed with saline solution to a defined volume of 250 ml and was infused over a 1 h period. Continuous drug administration was ensured by drop counting by the investigator.

### Local block

Ten minutes after the initial sedation bolus was administered, the maxillary or mandibular nerve was blocked with 0.4 mg/kg bwt lidocaine (Lidocainhydrochlorid 2%)^7^ as described elsewhere [18]. Another 10 min later the mouth gag was introduced and the surgery was started (20 min after the beginning of sedation). The adequacy of the local block was tested by utilizing the gingival separator and the molar spreader. If horses reacted to this manipulation, the local block was assessed as inadequate and the nerve block was repeated. Oral extraction of the cheek teeth was performed in a standardized manner as described by Tremaine [19]. After completion of the extraction, the intravenous catheter was removed and horses were placed back in their stalls.

### Measurements

All horses were assessed by one investigator who was blinded to the applied sedation protocol. Heart rate and RR were measured before the bolus application and in 10 min intervals thereafter. In addition the degree of ataxia, chewing and tongue activity as well as the head movement of the horses were graded by the investigator by means of a previously used scoring system (score 1–5; Table 1). If sedation was inadequate (scores for chewing/head movement/tongue activity ≥ 4), an additional bolus of romifidine (0.01 mg/kg bwt i.v.) was administered. After successful tooth extraction, the overall quality of sedation (overall behavior of the horses and degree of sedation) and quality of extraction (compliance of the horses to the surgical stimulus) was graded by the surgeon, who was blinded to the sedation protocol, using a visual analogue scale (VAS) from 1 to 10. One represented the best extraction or sedation quality and 10 described no signs of sedation and impossible cheek tooth extraction. All surgeries were performed by one of three surgeons, of whom one was a diplomate of the European Veterinary Dental College (EVDC) and two were final year residents of the European College of Veterinary Surgeons (ECVS). The relative head height was measured before and at 15 and 60 min after the initial sedation bolus. During relative head height measurements surgery was interrupted and horses were not handled.

**Table 1:** Scoring system by Hopster et al. [10] to evaluate the degree of ataxia, chewing, head movement and tongue activity of horses

Score	Ataxia	Chewing/head movement/tongue activity
1	No signs of ataxia, even load on the limbs	No chewing/head movement/tongue activity
2	Mild ataxia, mild swaying, occasionally leaning against the stocks	Occasional chewing/head movement/tongue activity
3	Moderate ataxia, constant leaning against the stocks, buckling of the limbs	Continuously mild chewing/head movement/tongue activity
4	Severe ataxia constant leaning against the stocks, buckling of the limbs	Continuously severe chewing/head movement/tongue activity
5	Recumbency	No manipulation possible

### Blood samples and cortisol analysis

Surgery was always performed in the morning between 9 a.m. and 12 p.m., to minimize the influence of the circadian cortisol rhythm [20]. Venous blood samples were collected from all horses three, two and 1 h as well as 15 min prior, and 15, 45, 60, 75, 90, 120, 150 and 180 min after bolus application (depending on the duration of the surgical procedure). During blood collection CRI was discontinued and 8 ml of blood were withdrawn from the intravenous catheter and discarded. Thereafter, another 8 ml of blood were obtained, transferred in serum tubes and the CRI was continued. At the end of the surgery, the CRI was stopped and two additional blood samples were taken after 20 and 120 min.

Each of the blood samples were incubated for 90 min at room temperature to allow complete clotting. Subsequently, the samples were centrifuged and the serum was stored at − 20°C prior to analysis.

Serum cortisol levels were assessed using a solid-phase, competitive, chemiluminescent enzyme immunoassay (Cortisol IMMULITE).^8^ The measuring range extended from 1 to 50 μg/dl with an analytical sensitivity reaching 0.2 μg/dl.

### Data analysis

Statistical analysis was performed with commercial software (SAS 9.2; SAS Inc., NC, USA). Gaussian distribution was tested by the Kolmogorov–Smirnov test and visual inspection of histograms. If data was not normally distributed non-parametric tests were used. Heart rate and RR were compared between groups and at different time points by one-way analysis of variance (ANOVA) with post-hoc Tukey test for multiple pair-wise comparisons. Differences in ataxia score were tested with permutation test and post-hoc Sidak-test for repeated measurements and evaluated for 100 min, when there were at least 6 horses in each group. The comparison of relative head height and sedation as well as extraction quality was determined by ANOVA. Head movement at different time points was analyzed by non-parametric ANOVA using the Kruskal-Wallis test followed by pair-wise comparison in between groups with Wilcoxon two sample test. Data of head movements were only analyzed as long as there were at least 5 horses in each sedation group left (this was the case until 60 min after beginning of surgery). Blood cortisol concentrations were compared by one way ANOVA and multiple pair-wise comparisons of the mean values. Significance level was set at *p* ≤ 0.05 and data are presented as mean +/− standard deviation.

## Results

The horses (29 Warmbloods, 7 ponies, 1 Thoroughbred and 3 Draft Horses) were between 3 and 27 years of age (mean 14 ± 6.4 years), weighing 313 up to 747 kg (mean 523 ± 108 kg). Detailed distribution of age, weight and gender between groups is listed in Table 2. There were no differences in distribution of gender and breed between groups. All 40 horses were healthy and their vital parameters were within normal limits.

**Table 2:** Distribution of age, weight and gender between groups

Sedation protocol	R	RB	RM	RK
Age	15 ± 6	13 ± 6	15 ± 6	14 ± 6
Weight	495 ± 74	496 ± 106	568 ± 135	534 ± 105
Gender	6 G, 4 M	4 G, 6 M	4 G, 6 M	4 G, 1 S, 5 M

Results for age and gender are presented as mean ± standard deviation

*G* gelding, *S* stallion, *M* mare, *Group R* Romifidine only, *group RB* Romifidine and Butorphanol, *group RM* Romifidine and Midazolam, *group RK* Romifidine and Ketamine

Heart rate and RR significantly decreased after the sedation bolus in all horses and remained significantly lower compared to baseline values during the whole time of sedation (Table 3).

**Table 3:** Values for the heart and respiratory rate before and during sedation

Sedation protocol	R	RB	RM	RK
Heart rate baseline	53 ± 22	44 ± 8	47 ± 11	44 ± 7
Heart rate sedation	29 ± 4^a^	29 ± 2^a^	31 ± 3^a^	28 ± 3^a^
Respiratory rate baseline	22 ± 6	19 ± 3	22 ± 12	21 ± 6
Respiratory rate sedation	13 ± 3^a^	13 ± 3^a^	12 ± 3^a^	14 ± 5^a^

Results are listed as mean ± standard deviation

*Group R* Romifidine only, *group RB* Romifidine and Butorphanol, *group RM* Romifidine and Midazolam, *group RK* Romifidine and Ketamine

^a^= significant difference to baseline measurement

A significant (*p* < 0.001) decrease in relative head height was detected in all horses 15 and 60 min after bolus application compared to the pre-sedation head height. Further, relative head height was significantly lower at 60 min compared to 15 min after bolus application. No significant difference between groups was found. Relative head height in group R decreased after 15 min to 65.25 ± 15.7% of the baseline values and after 60 min to 45.38 ± 14.25% of the pre-sedation values. In group RB and RM the results were 60.62 ± 13.64%, 37.76 ± 16.28% and 70.65 ± 12.62%, 45.18 ± 10.37%, respectively. Horses sedated with romifidine and ketamine showed a decrease of the relative head height to 60.01 ± 13.56% after 15 min and to 44.5 ± 12.73% after 60 min.

**Fig. 1:**
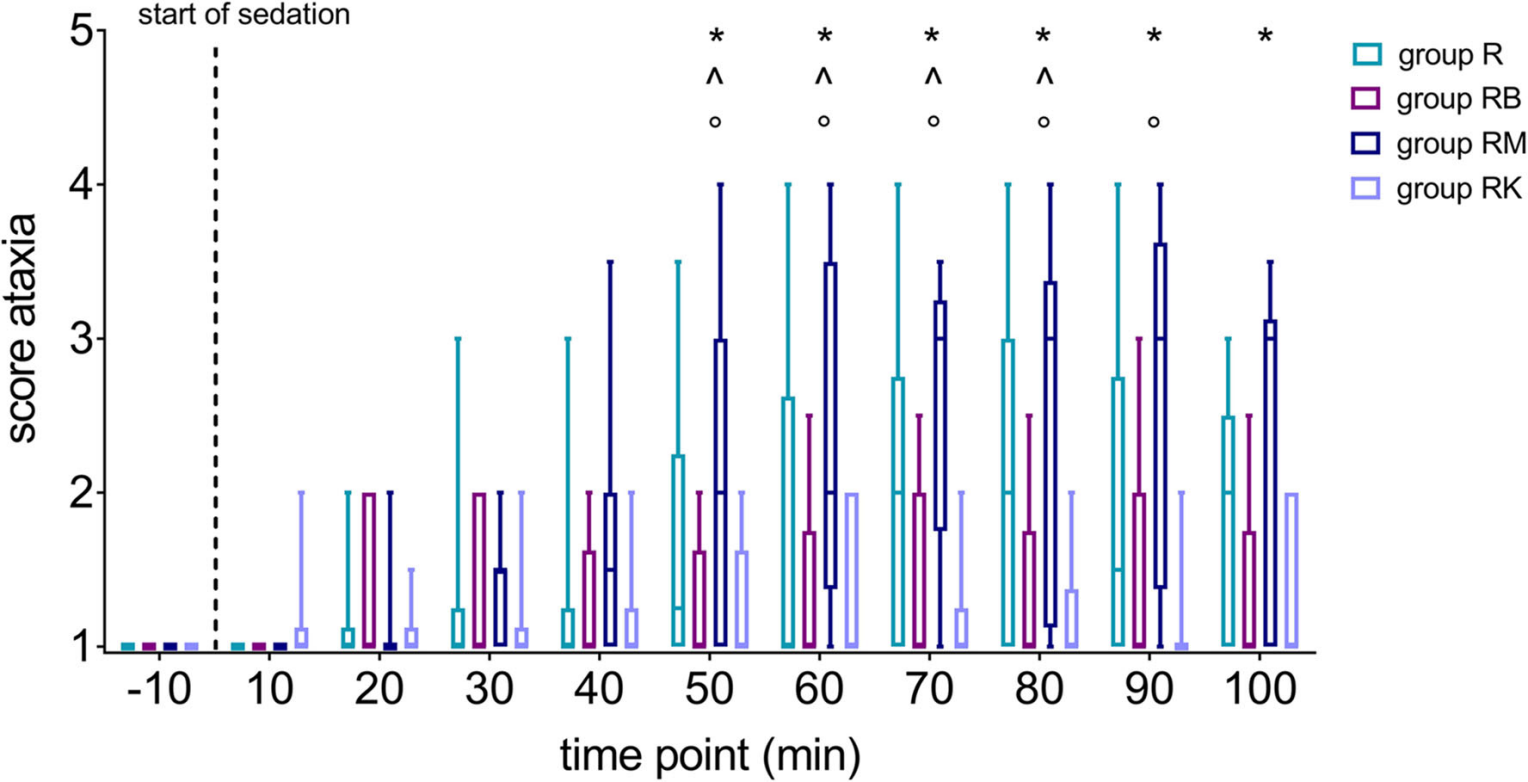
Degree of ataxia in 40 horses being sedated with romifidine (group R), romifidine and butorphanol (group RB), romifidine and midazolam (group RM) or romifidine and ketamine (group RK). Score values for ataxia are presented as qq-plots at the different time points. A score value of 1 stands for no signs of ataxia, whereas 5 describes recumbency (Table 1). Boxes and whiskers demonstrate interquartile range and the minimal/maximal values. * = significant difference between group RM and baseline; ^ = significant difference between group RB and RM; ° = significant difference between group RK and RM

Baseline measurements for ataxia were not significantly different between horses. Horses of group RM developed a significantly higher degree of ataxia during sedation compared to baseline and compared to horses of group RB and RK (Fig. 1).

Horses of group RM showed significantly less chewing than horses of group R (time point 30) and horses of group RB (time points 20, 30 and 50). Head movements of horses were not significantly different between treatments. Tongue activity scores were significantly lower in horses of group RM compared to groups R and RB at time points 30 and 50 min.

Numbers of romifidine boli necessary to deepen sedation are listed in Table 4. Time to the first top-up bolus was not statistically different among groups.

**Table 4:** Sedation and extraction quality for 40 horses using a visual analogue scale (1–10)

Sedation protocol	R	RB	RM	RK
Sedation quality	5 (1–9)	5 (2–7)	4 (1–6)	3 (1–5)^a^
Extraction quality	5 (1–10)	4 (1–7)	3 (1–8)	3.5 (1–9)
Top-up boli of romifidine	3.5 ± 2.1	2.3 ± 2.4	2.8 ± 3.2	2.2 ± 2.1
Horses with top-up boli	9/10	7/10	9/10	7/10
Time to first top-up bolus (min)	7.8 ± 10.6	20.6 ± 19.1	22.8 ± 15.8	17.8 ± 10.7
Adjusted CRI (mg/kg/h)	0.071	0.064	0.067	0.063

Scores are given as median (min - max). One represents the best extraction or sedation quality and 10 describes no signs of sedation and impossible cheek tooth extraction. The mean and standard deviation of required top up boli of romifidine (0.01 mg/kg bwt i.v.) for each group is also listed as well as the number of horses that needed additional sedation. Additionally the time to the first bolus application and the adjusted constant rate infusion (CRI) are mentioned

*Group R* Romifidine only, *group RB* Romifidine and Butorphanol, *group RM* Romifidine and Midazolam, *group RK* Romifidine and Ketamine

^a^= significant difference to group R

The sedation quality was considered to be significantly better in group RK compared to group R (Table 4). Median values for extraction quality are also listed in Table 4. In two horses of group R tooth extraction was not possible, because repeated romifidine boli failed to prevent defensive movement and did not enable surgery. The results of the evaluations of these two horses were included until the CRI was stopped and surgery was continued under general anesthesia. No significant differences in extraction quality between groups were detected.

### Cortisol analysis

**Fig. 2:**
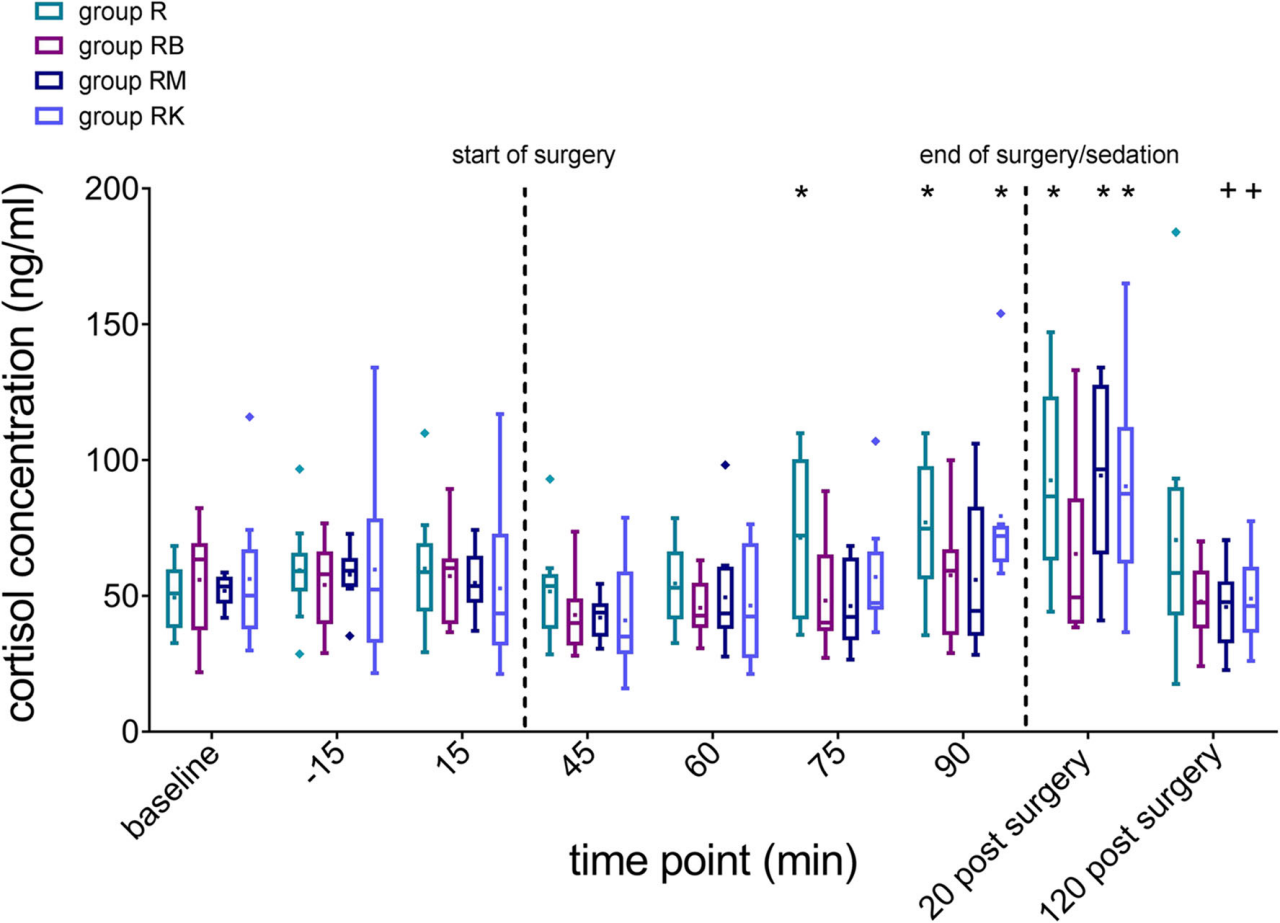
Serum cortisol concentrations (ng/ml) of 40 horses before, during and after cheek tooth extraction. Cortisol values are presented as qq-plots at different time points. Boxes and whiskers demonstrate interquartile range and standard deviation. Means are presented as points; values exceeding 1.5 times the interquartile range are presented as rhombus’. Group R= Romifidine only; group RB= Romifidine and Butorphanol; group RM= Romifidine and Midazolam; group RK= Romifidine and Ketamine. * = significantly different to baseline values; + = significantly different to the previous time point

Baseline measurements at 180, 120, 60 and 15 min before sedation revealed no statistically significant differences in serum cortisol concentrations between groups. Baseline concentrations were 49.34 ± 12.37 ng/ml, 55.95 ± 19.35 ng/ml, 51.9 ± 6.2 ng/ml and 56.27 ± 25.72 ng/ml in group R, group RB, group RM and group RK, respectively. In group R a significant increase above baseline was noticed 75 and 90 min after bolus application (Fig. 2). In group RK a significant increase in serum cortisol concentrations was detected 90 min after bolus administration compared to baseline measurements. No significant variations in cortisol concentrations were detected in groups RB and RM during sedation and tooth extraction.

Twenty minutes after discontinuing drug infusion, serum cortisol values in group R, RM and RK increased significantly above baseline values. In groups RM and RK cortisol concentrations returned to baseline concentrations 120 min after surgery. Horses of group RB showed no significant changes in cortisol levels during the postoperative period. Overall, cortisol concentrations were not significantly different among groups.

## Discussion

The four applied sedation protocols were able to produce chemical restraint for cheek tooth extraction in horses, except for two horses that were sedated with romifidine alone. However, in each group additional romifidine became necessary to perform dental surgery. For future clinical settings, an adjusted romifidine dosing regime is recommended.

In all horses the sedation resulted in a decrease in HR and RR, which is a well-known side effect of alpha-2-agonists [4, 21]. One horse in group R developed a HR below 20 beats per minute after receiving a total of six additional romifidine boli, which supports the fact that alpha-2 agonists mediate their cardiovascular depression in a dose-dependent manner [22]. There was no significant difference in HR between groups despite the trend in group R towards needing a higher total romifidine dose. This can be explained by the overall relatively low dose of romifidine that was administered.

Relative head height has been used as an indirect measurement for depth of sedation in horses [3]. Reduction in relative head height was evident under all of the four different protocols without differences between groups. Horses receiving romifidine and butorphanol showed a tendency towards carrying their head lower compared to the other groups. This might be due to synergistic effects of butorphanol on sedation with romifidine [2, 3]. On the other hand, an increase in relative head height caused by the central excitement induced by midazolam [23] and ketamine [24] cannot be ruled out in groups RM and RK.

A side effect of alpha-2 agonists is dose-dependent ataxia [5, 21]. Romifidine produces less ataxia compared to other alpha-2 agonists [5, 21], representing a considerable advantage for standing procedures. Horses in group R, RB and RK showed a satisfying ability to stand stable in the stocks. In contrast, horses sedated with romifidine in combination with midazolam were significantly more ataxic than horses in group RB and RK. Three horses of group RM even displayed severe ataxia with a total score of 4. The severe ataxia started about 40 min after bolus administration and continued until the end of CRI. The main reason for this increased ataxia is the skeletal muscle relaxation induced by midazolam [25]. Hubbell et al. [9] demonstrated that intravenous midazolam doses exceeding 0.1 mg/kg bwt in conscious horses can result in severe ataxia and might even lead to recumbency. Even though the applied midazolam dose in the present study was low compared to most studies, the concomitant romifidine administration should be considered as a potential enhancing factor. Special care should be taken in case of using midazolam in combination with alpha-2 agonists that produce more muscle relaxation than romifidine. Ataxia scores in horses only sedated with romifidine showed trends towards being higher than in horses that received combinations of romifidine with butorphanol or ketamine. The good standing stability in group RB and RK, nearly indistinguishable from baseline values, was unexpected. The addition of butorphanol to alpha-2 agonists is described to lead to more instability in standing horses [2, 3, 6]. In the current study, no significant increase in ataxia in group RB compared to the other groups was found. Iburg [26] described severe ataxia in horses placed in their stalls after administration of romifidine (0.05 mg/kg bwt i.v.) and ketamine (0.06 mg/kg bwt i.v.), which disagrees with the current results. Whereas Peterbauer et al. [12] and Lankveld et al. [13] detected only mild ataxia in horses in the first 5 min under ketamine CRI.

Successful cheek tooth extraction in standing horses is substantially influenced by chewing, head movement and tongue activity of horses. In our study, the combination of romifidine with midazolam was most effective in reducing chewing activity during surgery. This finding is probably related to the relaxation of the masticatory muscles caused by midazolam, which is in accordance with results of a previous study from the same working group [10]. In that study horses showed significantly less chewing activity when midazolam was added to the standard sedation protocol, consisting of romifidine (bolus 0.03 mg/kg bwt, CRI 0.04 mg/kg bwt/h) and butorphanol (bolus 0.01 mg/kg bwt, CRI 0.02 mg/kg bwt/h).

Overall, the intensity of head movements as an arbitrary defense reaction did not differ between treatment groups. The tendency towards higher score values at the beginning of sedation and surgery, especially in group R, indicated inadequate dosing for this invasive procedure. After additional top-up boli of romifidine the head movements were reduced. Although our dose of romifidine was in accordance with a previous study [10], a higher dosed initial bolus of romifidine would have been beneficial for this type of procedure.

Tongue activity was more intense in group R. This might be related to the site of action of alpha-2 agonists. Muscle relaxation as well as ataxia are mediated via inhibition of alpha-2 receptors in the spinal cord [27], whereas the tongue muscles are innervated by the hypoglossal nerve [28] and therefore are less affected by romifidine. The addition of butorphanol or ketamine did not significantly reduce tongue activity. In contrast, horses sedated with romifidine and midazolam showed significantly less tongue movements, which can be explained by the centrally acting midazolam mediating muscle relaxation via stimulation of GABA_A_-receptors [8].

Horses in group R showed trends towards receiving the largest number of top-up boli of romifidine. This resulted in an adjusted romifidine CRI of 0.071 mg/kg bwt/h compared to 0.064 mg/kg bwt/h in group RB, 0.067 mg/kg bwt/h in group RM and 0.063 mg/kg bwt/h in group RK. However, the larger amount of romifidine failed to gain better scores for measured head parameters compared to the other sedation protocols. Horses under romifidine midazolam sedation required on average 20% less additional romifidine boli than horses of group R. Reduced chewing and tongue activity improved the surgical conditions for cheek tooth removal and permitted a lower depth of sedation in combination with excellent extraction quality. Sedation quality was scored as “good”, but the lack of sedative and analgesic actions of midazolam and more pronounced ataxia might have influenced assessment. The combination of romifidine with butorphanol reduced the required top up boli by 34% compared to group R, and only 7 of 10 horses needed additional top ups. This can most likely be attributed to the synergistic actions on analgesia and sedation level [2, 29]. Horses under romifidine ketamine sedation needed the least additional romifidine and had the lowest and thereby the best scores for sedation and extraction quality. Ketamine has been shown to improve and prolong the analgesic effect of romifidine in horses [26]. This can explain the enhanced tolerance for manipulation in the mouth. Most horses received the additional romifidine boli immediately at the beginning of surgery, regardless of the sedation protocol, again indicating an inadequate initial sedation bolus. Marly et al. [30] administered a romifidine bolus of 0.08 mg/kg bwt followed by a CRI of romifidine 0.03 mg/kg bwt per hour for dental examination and treatment in horses. Sedation quality and depth was described as satisfying in their study, corroborating that a larger initial bolus should be used.

Another aim of the present study was to evaluate stress levels of horses before, during and after surgery by analyzing blood cortisol concentrations [17] at different time points. The baseline cortisol values were similar across all treatment groups. Neither sedation nor the beginning of the surgical procedure did result in changes in cortisol levels. The cortisol values increased in group R and RK 75 min and 90 min after the initial sedation bolus, respectively. This was surprising because tooth extraction was expected to be a strong stimulus to trigger the hypothalamic-pituitary-adrenal axis [15, 17]. However, one should take into account that alpha adrenoreceptors are widely spread within the *nucleus paraventricularis*, which is part of the hypothalamus [31]. Activation of presynaptic alpha-2 adrenoreceptors by alpha-2 agonists, such as romifidine, can result in inhibition of noradrenergic neurotransmitters leading to diminished cortisol concentrations as described in horses after application of clonidine or detomidine [32, 33]. The horses included in this study also received meloxicam pre-operatively, which may reduce the pain level in horses. In horses of group R, cortisol levels increased during tooth extraction reaching significant differences compared to baseline at the end of the procedure. We conclude that romifidine sedation alone failed to prevent the surgically induced stress response in horses, whereas sedation protocols RB and RM successfully suppressed this stress response. The reason for the lower cortisol levels in group RB might be the analgesic action of butorphanol and an increased depth of sedation. In addition, a direct inhibitory effect of opioids on the hypothalamus is described [34]. Such a direct inhibitory effect is also described for benzodiazepines [35] and might explain the low cortisol levels during sedation with romifidine and midazolam. Horses in group RK showed elevated cortisol values during surgery. These results were unexpected, considering the beneficial analgesic effect of ketamine and the good sedation and extraction evaluations of this protocol. However, ketamine can induce excitement of central nervous system [24] and can also selectively increase vasopressin concentrations within the hypothalamus and consequently trigger the hypothalamic-pituitary-adrenal axis, which in turn leads to increased cortisol release [36].

Twenty minutes after ceasing the CRI a significant increase in serum cortisol concentrations was detected in all treatment groups except for group RB. The inhibitory effect of romifidine on the hypothalamus might be reduced and stress responses could again be triggered. However, the duration of the sedative effect of romifidine is long in horses. A single dose of romifidine (0.04 mg/kg bwt) produces sedation for about 75 min [37], whereas in horses being sedated with a romifidine CRI (0.03 mg/kg bwt/h i.v.) sedation lasted for about 60 min after discontinuing the CRI [3]. Therefore it is likely that horses in the present study were still sedated 20 min after termination of the CRI. However, serum concentrations of romifidine might still have been too low for an effective inhibition of the hypothalamic-pituitary-adrenal axis. Furthermore, the analgesic effects of alpha-2 agonists require a higher plasma level than is necessary for sedation [38]. Therefore, it is possible that the horses had greater pain as the plasma levels of romifidine dropped despite residual sedation. Horses receiving butorphanol did not show any increased cortisol levels within the postoperative observation period. Butorphanol enhances and prolongs the analgesic and sedative properties of romifidine [2]. The last blood sample was taken 2 h postoperatively. At this time point all cortisol values were back to baseline except in group R, where cortisol levels were still elevated. Duration and intensity of cortisol elevation depend on the strength of the stress factor [15]. Therefore, it can be concluded that cheek tooth extraction was more distressing for the horses sedated with romifidine alone compared to the horses receiving butorphanol, midazolam or ketamine additionally.

A limitation of the study was the variability in duration of the procedure and the drug infusion time which is inherent to clinical trials. In case of incomplete maxillary or mandibular nerve blocks, the nerve infiltration had to be repeated before surgery could be continued, thereby leading to delays. The variable duration of the surgical procedure as well as ineffective anaesthesia resulting in varying levels of pain might have influenced the cortisol concentrations. Duration of surgery ranged from 108 min in group R to only 71 min in group RK. For the intraoperative measurements, we included only the first 60 min of surgery, however for the postoperative measurements the differences in duration of surgery could have had an impact on pain and stress evaluations.

Another limitation was the potential influence of the romifidine to up boli on the results for extraction and sedation qualities. It is most likely that in some horses a successful extraction would have failed without using additional romifidine application, which would have led to clearer results. But it must be accounted that the study was performed under clinical settings and therefore risking unsuccessful extraction was not an option.

The fact that all horses entered the treatment room and the stocks before sedation, can be considered as a limitation also. Depending on the character of the individual horse this might have already caused different levels of stress. However, no differences in cortisol levels were observed between samples taken in the stable or in the stocks. Administration of the primary sedation bolus before entering the treatment room and the stocks, or even when the horses are still in their stables, might be beneficial and is recommended for further trials. In future studies the recovery period and postoperative pain should be evaluated, which was not part of the present study.

## Conclusions

The combination of romifidine with midazolam facilitated oral manipulations in horses although higher levels of ataxia can be expected. An improvement of sedation quality was also obtained by adding ketamine instead of midazolam. Standing sedation for cheek tooth removal with romifidine alone led to increased stress levels, whereas the addition of butorphanol inhibited cortisol elevations during and after surgery.

## Abbreviations

ANOVA: Analysis of variance
CRI: Constant rate infusion
Group R: Horses sedated with romifidine alone
Group RB: Horses sedated with romifidine and butorphanol
Group RK: Horses sedated with romifidine and ketamine
Group RM: Horses sedated with romifidine and midazolam
HR: Heart rate
i.v.: Intravenous
RR: Respiratory rate
VAS: Visual analogue scale

## Footnotes

1 Vygon, Ecouen, France

2 Boehringer Ingelheim GmbH, Ingelheim

3 Rösch & Associates Information Engineering GmbH, Frankfurt am Main

4 CP-Pharma GmbH, Burgdorf

5 B. Braun Melsungen AG, Melsungen

6 Vétoquinol GmbH, Ravensburg

7 bela-pharm GmbH & Co. KG, Vechta

8 Siemens Medical Solutions, Bad Nauheim
